# GeneMill: A 21st century platform for innovation

**DOI:** 10.1042/BST20160012

**Published:** 2016-06-09

**Authors:** James R. Johnson, Rosalinda D'Amore, Simon C. Thain, Thomas Craig, Hannah V. McCue, Christiane Hertz-Fowler, Neil Hall, Anthony J.W. Hall

**Affiliations:** * GeneMill, Institute of Integrative Biology, University of Liverpool, Liverpool, L69 7ZB, U.K.

**Keywords:** automation, DNA cloning, DNA synthesis, metabolic phenotyping, reactor scale-up, Synthetic Biology

## Abstract

GeneMill officially launched on 4th February 2016 and is an open access academic facility located at The University of Liverpool that has been established for the high-throughput construction and testing of synthetic DNA constructs. GeneMill provides end-to-end design, construction and phenotypic characterization of small to large gene constructs or genetic circuits/pathways for academic and industrial applications. Thus, GeneMill is equipping the scientific community with easy access to the validated tools required to explore the possibilities of Synthetic Biology.

GeneMill is part of the wider Centre for Genomic Research (CGR) at University of Liverpool, which is a dedicated centre that facilitates academic and industrial access to state-of-the-art sequencing technologies for researchers worldwide. The success of the CGR over the past 10 years has led to further investment and development, now including Synthetic Biology. GeneMill was originally funded by RCUK in 2014 as one of the five UK DNA Foundries to set up a high-throughput DNA fabrication facility. Synthetic Biology is built on the principle of iterative cycles of *Design-Build-Test-Learn* and hence we have since developed significantly to include phenotyping platforms to close that loop (Figure 1). Thus, projects benefit not only from rapid sequence-perfect DNA construct/library synthesis, but also from the ability to assay the functionality of the synthesized DNA in the context of multiple hosts/chassis and culture conditions.

**Figure 1:**
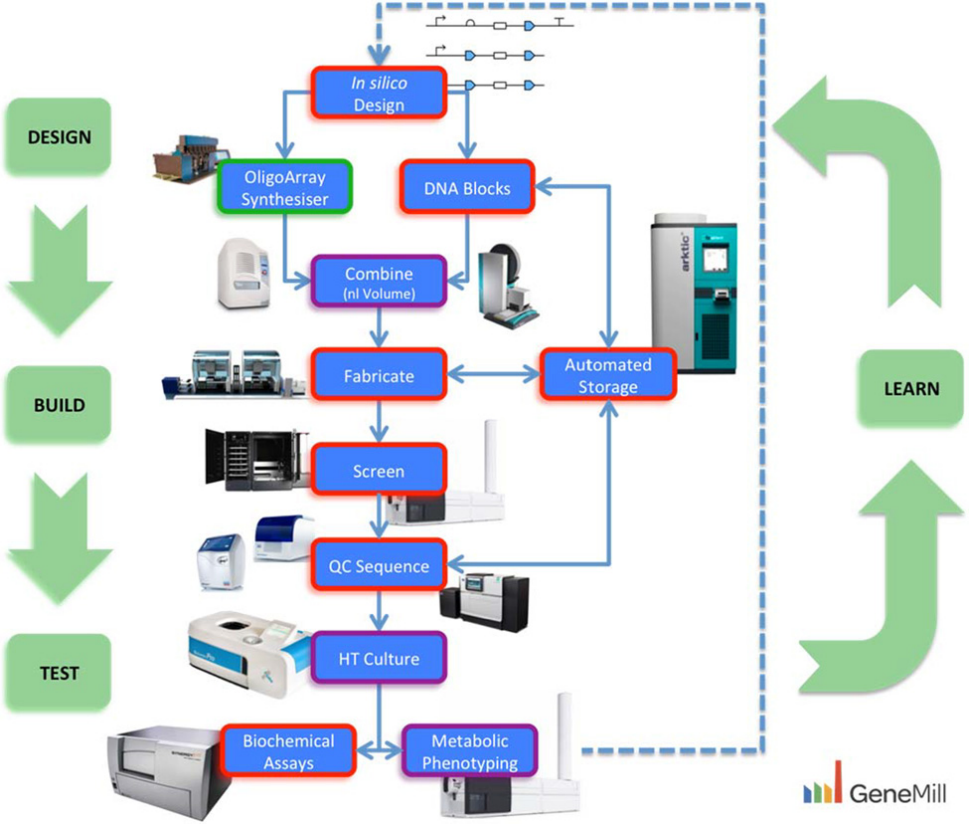
GeneMill overview DNA constructs are designed *in silico*. Functional parts are synthesized or retrieved from the automated storage and then combined at the nl scale with automated liquid handlers. DNA assembly reactions and transformations are performed, colonies picked and positive clones are taken forward for sequencing. Constructs are then assayed for function by small-scale culture or high thoughput (HT) micro-fermentation before being passed on for biochemical assays or metabolite analysis. Data can then be used to assess the function of the construct and/or host and potentially taken on for further design and optimization. Text Box borders indicate funding source; Red=GeneMill DNA Foundry, Purple=Assay Development Platform, Green=NextGen DNA Synthesis.

## Design

The focus and goal of GeneMill is to remove the complexity from the end user. DNA constructs can be designed by function rather than, as is traditional, by DNA sequence. Thus, the user simply needs to know the desired outcome of the designed construct, not the exact nucleotide sequence. Standardization and detailed annotation/ characterization of functional DNA parts (promoters, ribosome-binding sites, tags, terminators etc.) are key to achieving this goal [1]. GeneMill is working with other SynBio centres and the wider scientific community to develop consistent nomenclature and annotation rules on function and efficacy so that these parts can be designed, shared and used interchangeably throughout the community [2]. This takes a large step to reducing the current levels of variability in data produced from one laboratory to the next, encourages use of standard defined components and prevents the reinvention of ideas across different research groups. A repository of these functional parts is being produced and housed within GeneMill and will be accessed through a design portal, which will be available online in the coming months (Figure 1). This will take the form of a web-based graphical user interface, where users will also be able to track the progress of the project through the same online interface. Novel or custom sequences can also be requested here, as users will not be limited to predefined parts which could be added to GeneMill's growing parts repository. We will continue to work with academics from various research communities to build up a large repertoire of defined parts, which will benefit their community as a whole, increase impact and provide a lasting legacy [3]. Additionally, for non-community led projects, we have in-house established bioinformatics expertise in metagenomics, transcriptomic analysis and data mining to complete an end-to-end pipeline from pathway discovery to synthetic design. Industrial collaborators who wish to have private collections are also accommodated, and these will not be shared with the wider scientific community.

## Build

We take the final *in silico* DNA construct design and split it into its constituent parts. We have partnered with Twist Bioscience and GeneArt (Thermo Fisher Scientific) for DNA synthesis, which will produce the individual parts. In addition, we are working as part of a consortium to develop methods for higher fidelity oligonucleotide synthesis. All blocks and complete constructs will be stored in individually 2D-barcoded tubes, placed in an automated TTP Labtech Arktic −80°C freezer. Although this system has a small footprint, it holds >140000 tubes, storing and retrieving each one quickly and securely. Constituent parts are assembled together to form complete DNA constructs. To maximize efficiency of the assembly process, we are using ultra-low volume liquid handlers; the Labcyte Echo liquid handler uses acoustic energy, whereas TTP Labtech Mosquito liquid handlers use positive displacement tips to transfer nanolitre-scale volumes. This increases speed, reduces reagent volumes and eliminates cross-contamination. We are working closely with other U.K. Foundries to optimize these nanolitre-scale methods [4]. Standardization across multiple sites of these fundamental processes allows sharing of methods as well as DNA parts. A liquid handling robotics system, based around two CyBio FeliXs is then used to fabricate the final construct using high efficiency enzymatic assembly techniques [5]. This robot can perform automated thermal cycling and incubations to automate the transformation and plating of bacteria. A standalone K-Biosystems K6-2 system then identifies and picks colonies for culture and PCR-based QC via automated electrophoresis on Qiagen and Agilent instruments. DNA constructs pass through a stringent final QC stage where their sequences are verified. As part of the CGR, GeneMill has access to Next Generation sequencing technologies as well as long-read (>30 kb) single molecule sequencing allowing validation of sequence fidelity and assembly process.

The ability to automate the workflow described above guarantees reproducibility and standardization of DNA construct fabrication. It also means that protocols can be shared between sites and allow an identical workflow to be carried out. This eliminates the inevitable variability introduced by human scientists and removes the ‘art’ from molecular biology, reducing the time spent on cloning and allowing more of the investigators’ time to be spent on answering the important scientific questions [6].

## Test

As mentioned above, GeneMill not only provides an accessible DNA fabrication facility, we are also working closely with the Edinburgh Genome Foundry to develop pipelines for phenotypic analyses of novel proteins or synthetic pathways. From small-scale protein purification and enzyme prototyping to high-throughput micro-fermentation and metabolomics, GeneMill has an array of testing methodologies available. The RoboLector from M2P-Labs, for example provides a unique automated micro-fermentation system combining the high-throughput fermentation and online monitoring capabilities of the micro-bioreactor with automated sampling and control from the liquid handling robot. The online acquisition of relevant bioprocess data including biomass, fluorescent tag expression and culture oxygenation, allows precise and reliable bioprocess development, strain characterization, media optimization, direct up-scalability and clone screening in short time frames [7,8].

Metabolites of different samples, from individual bacterial colonies to cultures from a time course, can be analysed in great detail with our MS facilities. We have partnered with Agilent Technologies to equip GeneMill with the tools to examine a full range of metabolites. LC, GC or supercritical fluid chromatography (SFC) instruments upstream of an ion mobility-quantitative time-of-flight mass spectrometer (IM-QTOF MS) optimized for metabolic fingerprinting give us the ability to accurately identify metabolites of any type from complex sample mixtures [9]. Additionally, standard liquid chromatography-coupled mass spectrometry (LCMS) and gas chromatography-coupled mass spectrometry (GCMS) systems are available. These powerful techniques are key as they allow detailed interrogation and optimization of the fitness of each engineered strain or synthetic clone, from individual enzymes to entire synthetic pathways.

## Learn

Data gained from the techniques above can then be used to optimize the construct or host cell. For example MS data can be mapped to known biochemical pathways and combined and overlaid with other types of OMIC data to build up a detailed overview of cellular processes. We can then see the synthetic component as part of the whole system, rather than in isolation. Hosts as well as DNA constructs can then be optimized further.

The focus of GeneMill and the described workflow is available to the scientific community. Projects undertaken include the following: DNA fabrication, (plasmids, libraries and genome editing tools), expression optimization and designer proteins, enzyme prototyping, culture optimization, bioreactor scale-up and metabolic fingerprinting. Projects are managed from initial discussion to completion. Services are performed by postdoctoral GeneMill staff or by individual users gaining access to equipment and expertise in GeneMill labs (‘residential’). Alternatively, for larger projects, a dedicated member of staff may be recruited specifically to handle the project. We are also keen to work with whole communities to help build sets of parts for a specific organism or technology. These can then be delivered via the GeneMill as bespoke constructs.

## Abbreviations

CGR: Centre for Genomic Research

## References

[1] Quinn J.Y., Cox R.S., Adler A., Beal J., Bhatia S., Cai Y., Chen J., Clancy K., Galdzicki M., Hillson N.J. (2015). SBOL visual: a graphical language for genetic designs. PLoS Biol.

[2] Patron N.J., Orzaez D., Marillonnet S., Warzecha H., Matthewman C., Youles M., Raitskin O., Leveau A., Farré G., Rogers C. (2015). Standards for plant synthetic biology: a common syntax for exchange of DNA parts. New Phytol..

[3] Engler C., Youles M., Gruetzner R., Ehnert T.-M., Werner S., Jones J.D.G., Patron N.J., Marillonnet S. (2014). A golden gate modular cloning toolbox for plants. ACS Synth. Biol..

[4] Kanigowska P., Shen Y., Zheng Y., Rosser S., Cai Y. (2016). Smart DNA fabrication using sound waves: applying acoustic dispensing technologies to synthetic biology. J. Lab. Autom..

[5] Casini A., Storch M., Baldwin G.S., Ellis T. (2015). Bricks and blueprints: methods and standards for DNA assembly. Nat. Rev. Mol. Cell Biol..

[6] Linshiz G., Stawski N., Goyal G., Bi C., Poust S., Sharma M., Mutalik V., Keasling J.D., Hillson N.J. (2014). PR-PR: cross-platform laboratory automation system. ACS Synth. Biol..

[7] Hemmerich J., Adelantado N., Barrigón J.M., Ponte X., Hörmann A., Ferrer P., Kensy F., Valero F. (2014). Comprehensive clone screening and evaluation of fed-batch strategies in a microbioreactor and lab scale stirred tank bioreactor system: application on Pichia pastoris producing *Rhizopus*
*oryzae* lipase. Microb. Cell Fact..

[8] Polli F., Meijrink B., Bovenberg R.A., Driessen A.J. (2016). New promoters for strain engineering of Penicillium chrysogenum. Fungal Genet. Biol..

[9] Zhang F., Guo S., Zhang M., Zhang Z., Guo Y. (2015). Characterizing ion mobility and collision cross section of fatty acids using electrospray ion mobility mass spectrometry. J. Mass. Spectrom..

